# The Role of TCD in Assessing Postoperative Collateral Development and Long‐Term Clinical Outcome in Moyamoya Disease

**DOI:** 10.1111/cns.70245

**Published:** 2025-03-18

**Authors:** Shuangfeng Huang, Songtao Pei, Yiqin Han, Jiali Xu, Lanjing Wang, Heguan Fu, Changhong Ren, Xunming Ji, Sijie Li, Cong Han

**Affiliations:** ^1^ Department of Neurology, Xuanwu Hospital Capital Medical University Beijing China; ^2^ Department of Neurosurgery Fifth Medical Center of Chinese PLA General Hospital Beijing China; ^3^ Department of Rehabilitation Medicine Beijing Shijitan Hospital Affiliated to Capital Medical University Beijing China; ^4^ Department of Neurosurgery First Medical Center of Chinese PLA General Hospital Beijing China; ^5^ Beijing Key Laboratory of Hypoxic Conditioning Translational Medicine, Xuanwu Hospital Capital Medical University Beijing China; ^6^ Department of Emergency, Xuanwu Hospital Capital Medical University Beijing China

**Keywords:** cerebral hemodynamics, collateral development, moyamoya disease, postoperative cerebrovascular events, revascularization, transcranial Doppler

## Abstract

**Aims:**

To explore the role of transcranial Doppler (TCD) parameters after encephaloduroarteriosynangiosis (EDAS) to identify collateral development in moyamoya disease (MMD) and assess the relationship between these collateral formations and long‐term postoperative cerebrovascular events.

**Methods:**

A retrospective analysis of 91 MMD patients who underwent EDAS. Patients were categorized into rich or poor collateral groups based on postoperative angiography. TCD was used to monitor changes in hemodynamic parameters pre‐and post‐surgery. The association between clinical outcome, TCD parameters, and the degree of collateral development was investigated.

**Outcomes:**

Ninety‐one patients were assessed, with 45 (49.0%) exhibiting rich collaterals and 46 (51.0%) showing poor collaterals. Over 2 years, the rich collateral group experienced significantly fewer cerebrovascular events than the poor collateral group (*p* = 0.041). Postoperative evaluations demonstrated significant improvements in hemodynamic parameters within the rich collateral group, including increases in peak‐systolic velocity (PSV), end‐diastolic velocity (EDV), and mean velocity (MV), alongside decreases in resistance index (RI) and pulsatility index (PI) (*p* < 0.05). An EDV cutoff of > 16.62 cm/s in the superficial temporal artery (STA) effectively identified collateral development, yielding an area under the curve (AUC) of 0.907. Additionally, multivariate analysis revealed a strong association between preoperative MV of the STA and collateral formation.

**Conclusion:**

TCD sonography is a non‐invasive modality essential for assessing cerebral hemodynamics after revascularization in MMD. Collateral development shown on angiography corresponds to hemodynamic changes reflected in TCD. The postoperative EDV of the STA was a vital indicator of effective collaterals. Patients with well‐developed collaterals were at a lower risk of long‐term cerebrovascular events post‐surgery.

## Graphical abstract

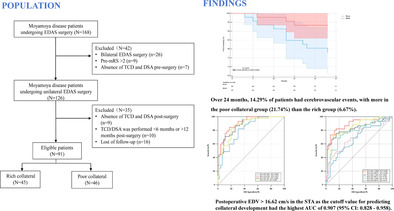

## 1 Introduction

Moyamoya disease (MMD) is a rare and progressive cerebrovascular disorder characterized by the stenosis or occlusion of the terminal internal carotid arteries, leading to the formation of a collateral vascular network at the base of the brain [1, 2, 3]. This condition predominantly manifests as strokes or transient ischemic attacks (TIAs), primarily due to insufficient cerebral blood flow [2]. Patients with symptomatic MMD are at a high risk of recurrent stroke, necessitating effective surgical interventions [4]. Various surgical procedures, including direct, indirect, and combination techniques, have been shown to restore blood supply to the cerebral hemisphere and reduce the risk of recurrent strokes [4, 5, 6, 7].

Indirect revascularization surgeries apply various grafts to promote neovascularization [8], and these grafts are commonly supplied by the ipsilateral superficial temporal artery (STA). Postoperative follow‐up emphasizes the evaluation of collateral formation from meningeal vessels and branches using angiography [9], which has been correlated with ultrasonographic changes in the STA [10]. Yeh et al. [10] found that increased end‐diastolic velocity (EDV) and decreased resistance index (RI) of the STA on ultrasound post‐surgery are sensitive and specific indicators of collateral development in patients undergoing indirect cerebrovascular reconstruction. Wang et al. [11] demonstrated that increasing the flow velocity of the STA by enlarging its inner diameter and enhancing the supply velocity significantly promoted neovascularization after revascularization procedures in patients with MMD. The supply of more blood to the brain by STA improved the patient's clinical symptoms and decreased the occurrence of adverse events [11]. Previous studies also indicated that an increase in postoperative collateral circulation is closely associated with patient prognosis [12], implying that monitoring these changes is crucial for improving outcomes and can guide the timing and feasibility of rescue revascularization surgeries.

Despite the promising outcomes of indirect revascularization procedures, a significant proportion of patients remain at risk for postoperative ischemic cerebrovascular events. Annual stroke rates were reported as high as 14.3% in adults and 2.3% in children with MMD [13]. In non‐operated patients, stroke risk is correlated with ultrasonographic parameters of the internal carotid artery and posterior cerebral artery; no specific parameter has been identified as associated with postoperative ischemic events [14]. The role of angiographic collateral grades and ultrasonographic parameters in predicting vascular events following indirect revascularization surgery remains unclear, particularly in the widely used encephaloduroarteriosynangiosis (EDAS) technique.

Given the current lack of a convenient, non‐invasive, and effective method for diagnosing postoperative collateral development in MMD, as well as the challenges in predicting long‐term cerebrovascular outcomes, there is a pressing need for innovative diagnostic approaches [15]. This study included 91 patients who underwent unilateral EDAS to analyze hemodynamic changes in the ipsilateral STA before and after surgery. Our objective is to assess the clinical significance of STA parameters measured by transcranial Doppler (TCD) sonography in identifying collateral development and its association with cerebrovascular events within 2 years post‐surgery. Ultimately, this research aims to guide more personalized and effective treatment strategies for patients with MMD.

## 2 Methods

### 2.1 Study Design and Participants

In this retrospective cohort study, we selected consecutive patients diagnosed with MMD who underwent EDAS surgery in the Department of Neurosurgery at the Fifth Medical Center of the PLA General Hospital from February 2019 to December 2021 to analyze the association between ultrasound parameters and postoperative efficacy in the ipsilateral cerebral hemisphere. This study was granted approval by the Research Ethics Committee of the Fifth Medical Center of the PLA General Hospital (ky‐2020‐9‐22), adhering to the ethical guidelines delineated in the Declaration of Helsinki. The study design adhered to the STROBE guidelines.

Participants who met the following criteria were included: (1) MMD was confirmed by digital subtraction angiography (DSA) based on the diagnostic criteria of the 2012 Research Committee on Moyamoya Disease (Spontaneous Occlusion of the Circle of Willis) of the Ministry of Health and Welfare of Japan [16]; (2) TCD sonography was performed within 1 week before surgery; (3) unilateral encephaloduroarteriosynangiosis (EDAS) was the sole surgical procedure performed; (4) follow‐up with DSA and TCD after surgery; (5) preoperative modified Rankin Scale (mRS) score ≤ 2; (6) follow‐up of clinical events for 2 years. Patients were excluded if they had any of the following: (1) no pre‐ or post‐operative DSA or TCD image; (2) unacceptable quality of the DSA or TCD image; (3) had undergone other surgical procedures.

### 2.2 Surgical Indications and Procedural Strategy

Our center primarily performed EDAS surgery for revascularization in the following cases: late‐stage MMD patients, young children, patients with improper donor or recipient vessels for direct bypass, and those with complicated medical conditions [17]. EDAS was considered based on guideline recommendations [4, 17], which entail an assessment of medical history, clinical symptoms, angiographic findings, and cerebral blood flow reserve capacity before surgery [18]. The primary surgical indications for MMD include: (1) symptoms of cerebral ischemia associated with disease, including TIA, infarction, cognitive decline, involuntary movement, headache, and seizures; (2) evidence of reduced cerebral blood flow reserve; (3) cerebral hemorrhage associated with MMD, while excluding other causes; and (4) the absence of surgical contraindications due to serious comorbidities affecting general anesthesia.

All patients underwent standard EDAS surgery [18], performed by an experienced neurosurgeon with over 15 years of experience (C.H.). As contralateral cerebral hemodynamics may change post‐unilateral surgery in MMD patients, only the hemisphere undergoing surgery was analyzed.

### 2.3 Evaluation of Collateral Establishment by Cerebral Angiography

DSA was conducted and interpreted by a seasoned neurologist. A 5F angiographic catheter was positioned at the C1 segment of the internal carotid artery, aligned with the second cervical vertebra, utilizing the Seldinger technique. The imaging protocol involved capturing four frames per second during the contrast injection. A power injector was employed to administer 5 mL of contrast medium at a rate of 3 mL per second, exerting a pressure of 300 psi/kg. This procedure was uniformly applied to all patients using a single‐plane angiographic system, the Artis zee floor by Siemens AG, Germany. Patients were categorized into two groups according to the degree of collateral development. Post‐surgical collateral vessel formation was classified into four distinct grades according to the extent of blood supply area coverage of the middle cerebral artery (MCA) on DSA: grade 0 indicated no clear coverage; grades 1, 2, and 3 corresponded to less than one‐third, one‐third to two‐thirds, and more than two‐thirds coverage, respectively. Grades 2 and 3 were classified in the rich collateral group, and lower grades were in the poor collateral group [19].

### 2.4 Ultrasonography

Ultrasonography was conducted using the Hitachi Aloka Arietta 70 ultrasound machine, equipped with a 2.0 MHz transducer, to assess the STA blood flow before and after the surgical procedure. The ultrasound examinations were uniformly performed by a single neurology ultrasound technician, who was blinded to the cerebral angiography outcomes to ensure unbiased measurements. The trunk of STA is in front of the auricle. The blood flow velocities were measured by pulsed‐wave Doppler within 60° of the STA trunk. The parameters included peak‐systolic velocity (PSV), EDV, mean velocity (MV), pulsatility index (PI), and RI of the STA. The MV, PI, and RI are calculated as follows: MV = (PSV‐EDV)/3 + PSV, PI = (PSV‐EDV)/MV, RI = (PSV‐EDV)/PSV. Given the potential correlation between these parameters and hemodynamic alterations, we meticulously analyzed both the absolute and percentage changes in values. The postoperative absolute changes (Δ) = postoperative value—preoperative value. The percentage changes (%) = absolute change/preoperative value × 100%. Accurately record ultrasonic parameters according to ultrasonography results.

### 2.5 Outcomes Assessment and Follow‐Up

Participants underwent DSA and TCD examinations assessment both at baseline and at 6–12 month follow‐up, and the interval between the two examinations was within 7 days. Postoperative collateral development was based on angiographic and ultrasonic parameters of postoperative follow‐up. Postoperative cerebrovascular events, including ischemic stroke, TIA, and hemorrhagic stroke events, were obtained through follow‐up within 2 years after surgery. Ischemic stroke is identified by new lesions on diffusion‐weighted imaging (DWI) or fluid‐attenuated inversion recovery (FLAIR). Hemorrhagic stroke is diagnosed with new high‐density lesions seen on DWI, FLAIR, or computed tomography (CT). TIA was defined as neurological symptoms that resolve within 24 h, showing no infarction on MRI or CT.

Postoperative clinical assessments were conducted systematically over 2 years, with monthly follow‐ups via clinical visits, telephone consultations, or review of medical records. During these visits, the patients were questioned about the occurrence of stroke or TIAs, revascularization surgery, current medications, cerebrovascular risk factor management, and any other discomfort. The collected data were recorded in the database.

### 2.6 Statistical Analysis

Continuous variables were presented as mean ± standard deviation or median with interquartile ranges (IQRs) in brackets. They were analyzed using either the Student's *t*‐test or the Mann–Whitney *U* test, depending on their distribution. Categorical variables were reported as counts of cases with percentages in parentheses and were subjected to either the Chi‐square test or Fisher's exact test, as appropriate. TCD parameter differences of the STA before and after revascularization surgery were analyzed using paired sample *t*‐tests and Student's *t*‐tests, emphasizing the comparison between rich and poor collateral development. Postoperative TCD parameters of the STA were assessed as potential indicators of collateral circulation development. Using the Matsushima grading based on angiography, we identified specific TCD parameters associated with rich collateral development. We quantified the sensitivities and specificities of these parameters through receiver operating characteristic (ROC) curves. Optimal cutoff values and the area under the curve (AUC) were determined to identify collateral development accurately. Univariate logistic regression analysis examined the correlation between preoperative TCD parameters of the STA and rich collateral development. Multivariate logistic stepwise regression analysis was performed to screen TCD parameters with *p* less than 0.05 in univariate analysis and confounding factors (Suzuki stages and age) confirmed by previous studies [20], to identify independent preoperative parameters related to rich collateral development. The cumulative incidence of cerebrovascular events from time to event was estimated using Kaplan–Meier survival analysis. Statistical analysis was conducted using SPSS (v24.0; IBM Inc.), complemented by the R programming language and R environment version 4.2.3 (http://cran.r‐project.org), with a significance level of *p* < 0.05.

## 3 Results

### 3.1 Patient Characteristics

A total of 91 MMD patients (91 hemispheres) were eligible for enrollment (Table 1 and Figure S1). The mean age was 42.68 ± 10.48 years, and there were 45 women and 46 men. The primary preoperative symptom observed was ischemic stroke, with Suzuki stage 4 being the most prevalent. DSA data were available for 91 hemispheres; 45 hemispheres were in the rich collateral group (grades 2, 3) and 46 hemispheres were in the poor collateral group (grades 0, 1), according to the postoperative Matsushima grading. There were no significant differences in age, sex, history of other risk factors, preoperative clinical symptoms, lesion type, Suzuki stage, preoperative mRS scores, or follow‐up period between the rich collateral group and the poor collateral group (*p* > 0.05; Table 1).

**TABLE 1:** Basic demographic data of patients with Moyamoya disease.

Variables	Total patients (*n* = 91)	Poor collateral (*n* = 46)	Rich collateral (*n* = 45)	*p*
Age (year), mean ± SD	42.68 ± 10.48	44.65 ± 10.56	40.67 ± 10.11	0.069
Male, *n* (%)	46 (50.55)	23 (50.00)	23 (51.11)	0.916
*Medical history*, *n (%)*				
Hypertension	52 (57.14)	26 (56.52)	26 (57.78)	0.904
Diabetes	12 (13.19)	9 (19.57)	3 (6.67)	0.069
Hyperlipidemia	31 (34.07)	14 (30.43)	17 (37.78)	0.460
Thyroid disease	7 (7.69)	5 (10.87)	2 (4.44)	0.449
Smoking	17 (18.68)	9 (19.57)	8 (17.78)	0.827
Drinking	10 (10.99)	2 (4.35)	8 (17.78)	0.087
*Preoperative clinical symptoms*, *n (%)*				
Ischemia	77 (84.62)	37 (80.43)	40 (88.89)	0.264
Hemorrhage	14 (15.38)	9 (19.57)	5 (11.11)	
*Lesion type*, *n (%)*				
Bilateral	63 (69.23)	29 (63.04)	34 (75.56)	0.196
Unilateral	28 (30.77)	17 (36.96)	11 (24.44)	
*Suzuki stages*, *n (%)*				
2	7 (7.69)	2 (4.35)	5 (11.11)	0.212
3	10 (10.99)	7 (15.22)	3 (6.67)	
4	48 (52.75)	27 (58.69)	21 (46.67)	
5	16 (17.58)	5 (10.87)	11 (24.44)	
6	10 (10.99)	5 (10.87)	5 (11.11)	
*mRS scores*, *n (%)*				
0	57 (62.64)	29 (63.04)	28 (62.22)	0.997
1	18 (19.78)	9 (19.57)	9 (20.00)	
2	16 (17.58)	8 (17.39)	8 (17.78)	
Postoperative follow‐up (month)	9.63 ± 2.12	9.31 ± 2.26	9.95 ± 1.95	0.153

Abbreviations: mRS, Modified Rankin Scale; SD, standard deviation.

### 3.2 Evaluation of Cerebral Blood Flow Changes

The results of the postoperative DSA revealed that 29 (31.87%) of the 91 patients exhibited grade 3 collateral formation, 16 (17.58%) exhibited grade 2, and the remaining 31 (34.07%) and 15 (16.48%) patients exhibited grade 1 and grade 0, respectively. Thus, 45 patients (49.45%) were classified into the rich collateral group, and 46 patients (50.55%) were classified into the poor collateral group.

### 3.3 Incidence of Postoperative Cerebrovascular Events

**FIGURE 1:**
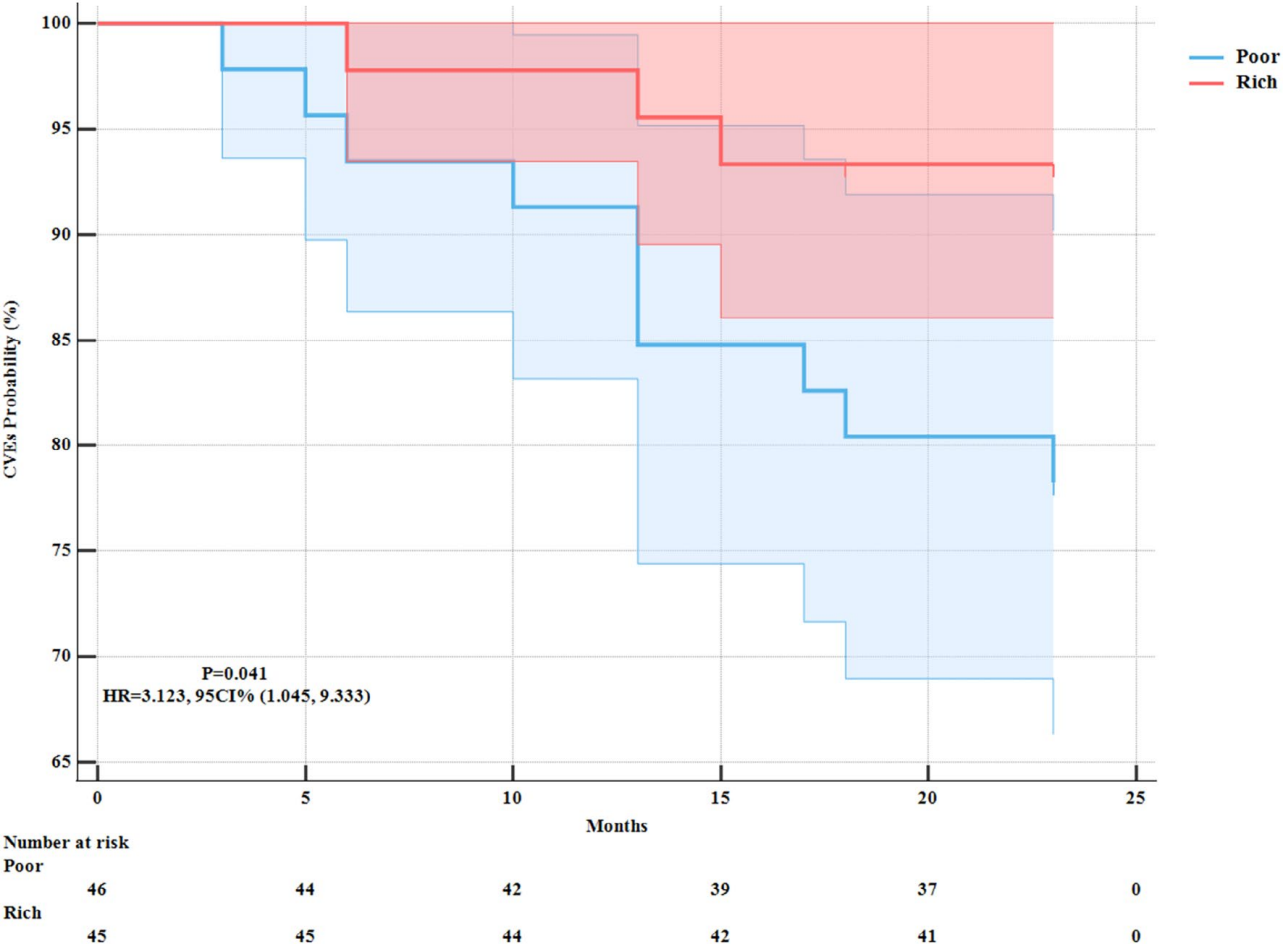
Cumulative incidence of cerebrovascular events (CVEs) at follow‐up. HR, hazard ratio; CI, Confidence Interval.

During a follow‐up period of 2 years, 13 patients (14.29%) experienced cerebrovascular events, including 4 (4.40%) ischemic strokes, 7 (7.69%) TIAs, and 2 (2.20%) hemorrhagic strokes. Three patients (6.67%) in the rich collateral group and 10 patients (21.74%) in the poor collateral group experienced cerebrovascular events (*p* = 0.041) (Figure 1).

### 3.4 Comparison of Ultrasound Parameters Before and After Surgery

The results showed that the overall mean PSV increased significantly after surgery, from 44.74 ± 12.32 cm/s to 52.61 ± 14.66 cm/s (*p* < 0.001). In the rich collateral group, the increase in mean PSV was even more pronounced, rising from 47.55 ± 12.34 cm/s to 60.42 ± 12.53 cm/s (*p* < 0.001). In the poor collateral group, however, the increase in mean PSV was not significant, with a modest increase from 42.00 ± 11.79 cm/s to 44.97 ± 12.47 cm/s (*p* = 0.086). The EDV of the entire patients showed significant improvement postoperatively, increasing from 10.68 ± 5.04 cm/s to 18.24 ± 9.55 cm/s (*p* < 0.001). The rich collateral group showed a greater increase in EDV from 11.67 ± 5.84 cm/s to 24.57 ± 8.64 cm/s (*p* < 0.001), while the poor collateral group also showed an increase, albeit to a lesser extent, from 9.71 ± 3.94 cm/s to 12.05 ± 5.49 cm/s (*p* = 0.002). Both the overall and rich collateral groups showed a more significant increase in postoperative MV (*p* < 0.001), compared to the poor collateral group (*p* = 0.010). In terms of PI and RI, both the overall and rich collateral groups showed significant reductions after surgery (*p* < 0.001). However, the poor collateral group showed a more modest decrease in these indices (PI: *p* = 0.003, RI: *p* = 0.002). Table 2 presents the complete data entries.

**TABLE 2:** Comparison of ultrasound parameters in MMD patients pre‐ and post‐surgery.

		Pre‐surgery	Post‐surgery	*t*	*p*
PSV	Overall	44.74 ± 12.32	52.61 ± 14.66	−5.057	< 0.001
	Rich	47.55 ± 12.34	60.42 ± 12.53	−5.299	< 0.001
	poor	42.00 ± 11.79	44.97 ± 12.47	−1.757	0.086
EDV	Overall	10.68 ± 5.04	18.24 ± 9.55	−8.243	< 0.001
	Rich	11.67 ± 5.84	24.57 ± 8.64	−10.053	< 0.001
	poor	9.71 ± 3.94	12.05 ± 5.49	−3.216	0.002
MV	Overall	22.03 ± 6.85	29.70 ± 10.72	−7.217	< 0.001
	Rich	23.63 ± 7.45	36.52 ± 9.55	−8.178	< 0.001
	poor	20.47 ± 5.89	23.02 ± 6.70	−2.674	0.010
PI	Overall	1.59 ± 0.30	1.25 ± 0.37	8.957	< 0.001
	Rich	1.58 ± 0.32	1.02 ± 0.23	12.256	< 0.001
	poor	1.60 ± 0.29	1.47 ± 0.34	3.101	0.003
RI	Overall	0.77 ± 0.07	0.67 ± 0.11	9.186	< 0.001
	Rich	0.76 ± 0.08	0.60 ± 0.08	12.692	< 0.001
	poor	0.77 ± 0.07	0.73 ± 0.09	3.248	0.002

Abbreviations: EDV, end‐diastolic velocity; MMD, moyamoya disease; MV, mean velocity; PI, pulsatility index; PSV, peak systolic velocity; RI, resistance index.

### 3.5 Postoperative STA Parameters: Rich vs. Poor Collateral Development

This study conducted a statistical analysis of hemodynamic indicators in the rich and poor collateral groups before and after surgery. The results indicated that the preoperative mean PSV was 42.00 ± 11.79 cm/s in the poor collateral group and 47.54 ± 12.34 cm/s in the rich collateral group, indicating a significant difference between the two groups (*p* = 0.031). The rich collateral group also exhibited higher average values in MV, specifically 23.63 ± 7.45 cm/s, compared to 20.47 ± 5.89 cm/s in the poor group (*p* = 0.027), indicating a notable difference in MV. There were no significant differences in EDV, PI, and RI between the two groups (*p* = 0.062, *p* = 0.674, and *p* = 0.599, respectively).

Postoperatively, the PSV in the poor collateral group increased to 44.97 ± 12.47 cm/s, while it significantly increased to 60.42 ± 12.53 cm/s in the rich collateral group, making the difference between the two groups even more significant (*p* < 0.001). Postoperative indicators such as EDV, MV, PI, and RI also showed significant differences between the two groups (*p* < 0.001).

From the perspective of absolute changes, the rich collateral group showed significantly greater changes in PSV, EDV, MV, PI, and RI compared to the poor collateral group (*p* < 0.001), further supporting the conclusion that surgery had a greater improvement effect on hemodynamic indicators in the rich collateral group. Additionally, in terms of percentage changes (%), the rich group also demonstrated significantly better improvements in PSV%, EDV%, MV%, PI%, and RI% compared to the poor collateral group (*p* < 0.001 or *p* = 0.005). Table 3 presents the complete data entries.

**TABLE 3:** Comparison of ultrasound parameters between the poor collateral group and rich collateral group for MMD.

	Poor collateral (*n* = 46)	Rich collateral (*n* = 45)	*p*
*Pre‐surgery*			
PSV	42.00 ± 11.79	47.54 ± 12.34	0.031
EDV	9.71 ± 3.94	11.67 ± 5.84	0.062
MV	20.47 ± 5.89	23.63 ± 7.45	0.027
PI	1.60 ± 0.29	1.58 ± 0.32	0.674
RI	0.77 ± 0.07	0.76 ± 0.08	0.599
*Post‐surgery*			
PSV	44.97 ± 12.47	60.42 ± 12.53	< 0.001
EDV	12.05 ± 5.49	24.57 ± 8.64	< 0.001
MV	23.02 ± 6.97	36.52 ± 9.55	< 0.001
PI	1.47 ± 0.34	1.02 ± 0.23	< 0.001
RI	0.73 ± 0.09	0.60 ± 0.08	< 0.001
△PSV	2.96 ± 11.44	12.87 ± 16.29	0.001
△EDV	2.34 ± 4.93	12.90 ± 8.61	< 0.001
△MV	2.55 ± 6.46	12.89 ± 10.57	< 0.001
△PI	−0.13 ± 0.29	−0.56 ± 0.30	< 0.001
△RI	−0.04 ± 0.07	−0.16 ± 0.08	< 0.001
PSV%	0.11 ± 0.31	0.35 ± 0.48	0.005
EDV%	0.34 ± 0.66	1.54 ± 1.66	< 0.001
MV%	0.17 ± 0.39	0.68 ± 0.74	< 0.001
PI%	−0.07 ± 0.18	−0.34 ± 0.16	< 0.001
RI%	−0.04 ± 0.10	−0.21 ± 0.10	< 0.001

Abbreviations: ΔEDV, change in the mean end‐diastolic velocity; EDV%, percent change in the mean end‐diastolic velocity; EDV, end‐diastolic velocity; ΔMV, change in the mean velocity; ΔMV%, percent change in the mean velocity; MMD, moyamoya disease; MV, mean velocity; ΔPSV, change in the mean peak systolic velocity; ΔPI, change in the pulsatility index; PI, pulsatility index; PI%, percent change in the pulsatility index; PI%, percent change in the resistance index; PSV%, percent change in the mean peak systolic velocity; PSV, peak systolic velocity; ΔRI, change in the resistance index; RI, resistance index.

### 3.6 Cutoff and AUC of Rich Collateral Development

**FIGURE 2:**
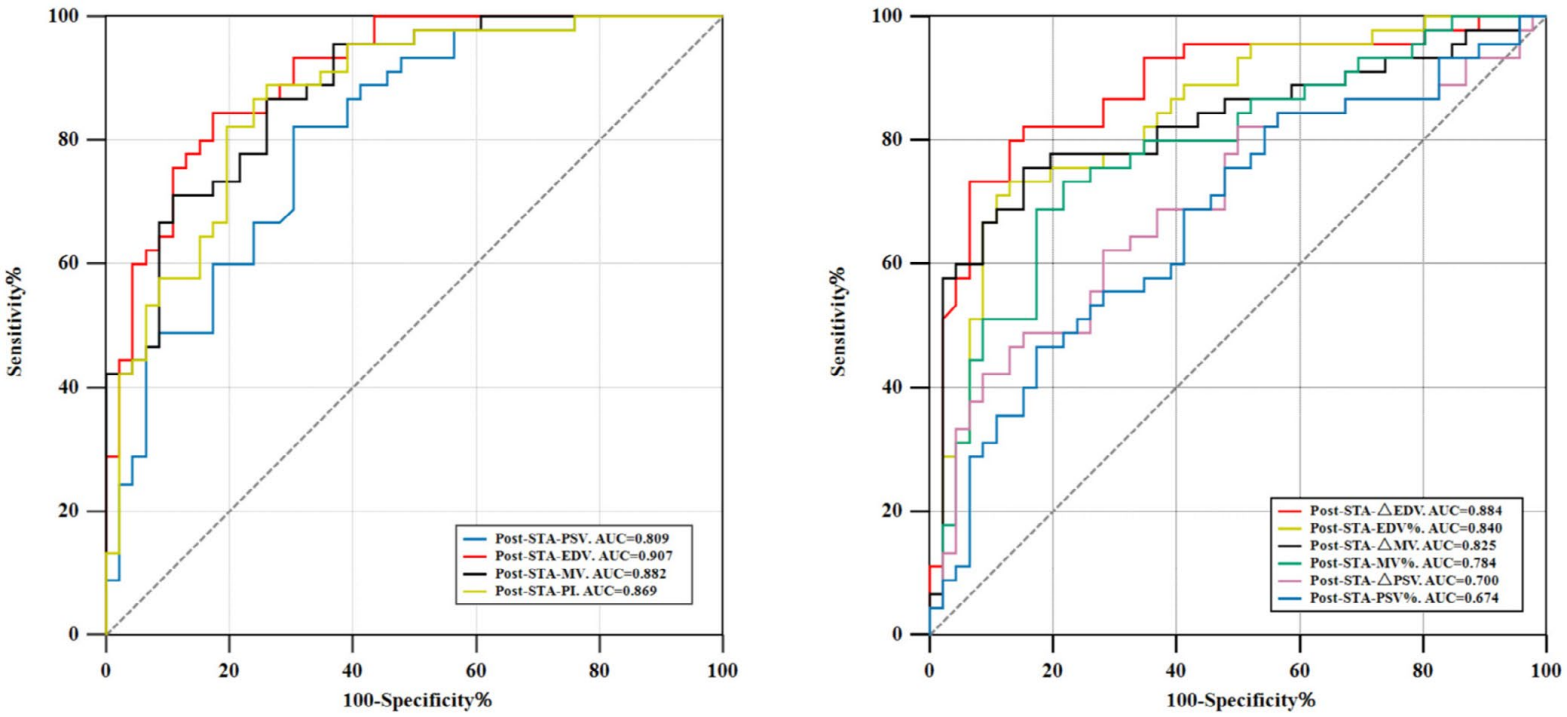
The cutoff values and AUC of ultrasound parameters for the collateral development post‐surgery. STA, Superficial Temporal Artery; PSV, peak systolic velocity; ΔPSV, change in the mean peak systolic velocity; PSV%, percent change in the mean peak systolic velocity; EDV, end‐diastolic velocity; ΔEDV, change in the mean end‐diastolic velocity; EDV%, percent change in the mean end‐diastolic velocity; MV, mean velocity; ΔMV, change in the mean velocity; ΔMV%, percent change in the mean velocity; PI, pulsatility index; AUC, Area Under the Curve.

Postoperative EDV > 16.62 cm/s in the STA as the cutoff value for identifying rich collateral development had the highest AUC of 0.907 (95% CI: 0.828–0.958) with 84.44% sensitivity, 82.61% specificity, 82.64% PPV, and 84.41% NPV (Figure 2). Postoperative MV values exceeding 27.50 cm/s demonstrated strong identification ability, with a sensitivity of 86.67%, a specificity of 73.91%, and an AUC of 0.882 (95% CI: 0.798–0.940). In the changes of the TCD parameters after revascularization, a postoperative absolute increase in EDV of more than 5.77 cm/s in the STA, as the cutoff value for identifying rich collateral development, had the highest AUC of 0.884 (95% CI: 0.800–0.942), with 82.22% sensitivity, 84.78% specificity, 84.11% PPV, and 82.95% NPV (Figure 2). Additionally, postoperative PSV, postoperative PI, postoperative RI, △PSV, △MV, PSV%, EDV%, and MV% in the STA also provide different degrees of predictive value (Table 4 and Figure 2).

**TABLE 4:** Predictive performance of ultrasound parameters for postoperative rich collateral development: cutoff values and AUC analysis.

	Cut off	Sensitivity (%)	Specificity (%)	AUC (95% CI)	PPV	NPV	*p*
Post‐STA‐PSV	> 50.22	82.22	69.57	0.809 (0.714–0.884)	72.59	79.97	< 0.0001
Post‐STA‐EDV	> 16.62	84.44	82.61	0.907 (0.828–0.958)	82.64	84.41	< 0.0001
Post‐STA‐MV	> 27.50	86.67	73.91	0.882 (0.798–0.940)	76.50	84.98	< 0.0001
Post‐STA‐PI	≤ 1.33	88.89	73.91	0.869 (0.782–0.931)	76.96	87.16	< 0.0001
Pre‐STA‐RI	≤ 0.70	88.89	73.91	0.869 (0.782–0.931)	76.96	87.16	< 0.0001
Post‐STA‐△PSV	> 8.68	62.22	71.74	0.700 (0.595–0.792)	68.34	65.95	0.0004
Post‐STA‐PSV%	> 0.29	46.67	82.61	0.674 (0.568–0.769)	72.46	61.25	0.0023
Post‐STA‐△EDV	> 5.77	82.22	84.78	0.884 (0.800–0.942)	84.11	82.95	< 0.0001
Post‐STA‐ EDV%	> 0.72	73.33	86.96	0.840 (0.748–0.908)	84.64	76.89	< 0.0001
Post‐STA‐△MV	> 8.48	75.56	84.78	0.825 (0.731–0.896)	82.95	77.97	< 0.0001
Post‐STA‐MV%	> 0.34	73.33	78.26	0.784 (0.685–0.863)	76.78	74.96	< 0.0001

Abbreviations: AUC, area under the curve; ΔEDV, change in the mean end‐diastolic velocity; ΔEDV%, percent change in the mean end‐diastolic velocity; EDV, end‐diastolic velocity; ΔMV, change in the mean velocity; ΔMV%, percent change in the mean velocity; MV, mean velocity; NPV, negative predictive value; ΔPSV, change in the mean peak systolic velocity; ΔPSV%, percent change in the mean peak systolic velocity; PI, pulsatility index; PPV, positive predictive value; PSV, peak systolic velocity; RI, resistance index; STA, superficial temporal artery.

### 3.7 Preoperative STA Parameters in Collateral Assessment

Preoperative STA parameters PSV and MV as the independent variables were incorporated into the logistic regression equation with rich collateral development as the dependent variable for univariate and multivariate analysis. After adjusting for patient age and Suzuki stage, multivariate analysis showed a significant correlation between preoperative MV of STA and the rich collateral development post‐revascularization (*p* = 0.032; OR = 1.07, 95% CI 1.01–1.15, Table S1).

## 4 Discussion

In this retrospective analysis of 91 patients undergoing cerebral revascularization surgery, our findings indicated a significant improvement in hemodynamics, particularly in patients with robust collateral formation. Angiographic evidence of collateral development corresponded with hemodynamic changes observed in TCD parameters. The current study showed specific postoperative ultrasonographic parameters at 6–12 months as identifiers of good collateral development. Notably, an EDV of the STA exceeding 16.62 cm/s or an absolute increase of 5.77 cm/s suggests good collateral formation. This good collateral development may positively influence postoperative prognosis, potentially reducing the risk of cerebrovascular events within 2 years after surgery.

In the present study, collateral vascularization after surgery was shown in 91 hemispheres, with 45 exhibiting a rich collateral vessel formation, categorized as grades 2 or 3. These results aligned with previous studies [21, 22]. The TCD findings from this study further substantiate the effectiveness of EDAS surgery, indicating a significant improvement in the perfusion of ischemic brain tissue in the rich group. This was also consistent with previous studies [23].

This study analyzed the differences in ultrasound parameters between patients with rich and poor collateral development. Our results indicated a significant increase in PSV, EDV, and MV in the group with rich collateral development post‐EDAS, compared to the group with poor collateral development. Currently, we observed a considerable decrease in PI and RI in the former group. These findings were indicative of enhanced blood flow and reduced vascular resistance in the STA of patients with rich collateral development, suggesting a more pronounced neovascularization response in the brain tissue. Longitudinal surveillance of cerebrovascular events has shown that an enhanced blood supply to the brain, mediated by the STA, was associated with reduced clinical symptoms and a lower likelihood of cerebrovascular incidents. Previous research supported this association by highlighting a connection between the extent of collateral vessel formation seen on cerebral angiograms following indirect revascularization surgeries and patient outcomes [24]. Assessing collateral vessel development is a vital indicator of surgical success and therapeutic efficacy. For patients with inadequate collateral vessels, additional interventions may be warranted to mitigate the risk of complications and optimize cerebral blood flow. These interventions could include enhancing cerebral blood flow through secondary salvage surgery, promoting collateral circulation with neuroprotective therapies like remote ischemic conditioning [17, 25, 26], and managing factors affecting collateral formation, such as blood homocysteine levels and blood sugar [27, 28]. Additionally, long‐term neuroimaging follow‐ups are conducted to monitor the progression of angiopathy [29]. The implementation of these strategies is pivotal in managing patients with compromised collateral circulation, intending to reduce the incidence of postoperative ischemic events and enhance patient outcomes.

The development of collaterals through neovascularization is the major mechanism in indirect revascularization surgery, aimed at alleviating cerebral hypoperfusion and preventing recurrent ischemic events. Our study indicated that patients exhibiting rich collateral development experience fewer cerebrovascular events over a 2‐year follow‐up compared to those with poor collateral formation. This finding is consistent with previous research, including Yeh et al. which demonstrated that ultrasonographic parameters of the ipsilateral STA at 3 months post‐surgery are independently associated with postoperative cerebrovascular ischemic events in MMD patients [30]. Notably, a lower RI in the ipsilateral STA correlates with improved collateral formation (Matsushima grade A or B), suggesting these ultrasound metrics could serve as valuable predictors for reducing postoperative vascular events [31]. Postoperative ischemic events emerge as the sole independent predictor of unfavorable outcomes after revascularization in MMD patients, underscoring the need for early prediction and timely intervention. The reported predictors for postoperative cerebrovascular events were limited to clinical and neuroimaging factors [32], our current study demonstrated that postoperative ultrasound parameters can effectively identify collateral development. Additionally, varying degrees of collateral formation significantly impact the occurrence of cerebrovascular events within 2 years post‐surgery. This underscores the advantages of ultrasound in assessing the complex hemodynamic changes due to neovascularization and progressive arterial stenosis, ultimately influencing clinical outcomes.

Previous studies have shown that STA parameters in ultrasound were valuable for evaluating the effectiveness of surgical procedures in promoting alternative blood vessel growth [11]. Our results showed that the EDV of the STA was the most responsive indicator of postoperative collateral development following revascularization surgery. Specifically, we found that a postoperative EDV exceeding 16.62 cm/s in the STA is a significant predictor for establishing collateral circulation. Moreover, when considering the absolute change in EDV post‐surgery, an EDV increase exceeding 5.77 cm/s in the STA also showed robust predictive power for collateral development. Yeh et al. [10] evaluated the ultrasonographic responses of the STA after indirect vascular reconstruction in 24 hemispheres, noting that an EDV increase of at least 13.5 cm/s indicated high sensitivity and specificity for assessing collateral development post‐surgery. In our study, we observed a rise in EDV greater than 5.77 cm/s with an AUC of 0.884, identifying good collateral establishment. In contrast to the cohort studied by Yeh et al. [10], which primarily included pediatric and young adult patients with a mean age of 17 ± 10.2 years, our investigation focused on an older demographic with an average age of 42.68 ± 10.48 years. Previous studies have demonstrated that pediatric patients typically exhibit more pronounced postoperative ultrasonographic changes, contributing to greater increases in PSV, EDV, and flow volume following surgery [33]. This age‐related difference may account for the smaller increase in EDV observed in our findings. Additionally, the different indirect revascularization techniques employed in the two studies may explain the variations in results. Our study focused on EDAS surgery, and other indirect revascularization techniques involved in the research above include Encephalo‐Myosynangiosis (EMS), Encephalo‐Periosteal‐Synangiosis (EPS), and combined EDAS with EPS and EMS [10]. Although all these surgeries used STA as a material for revascularization, prior research has indicated that the blood flow velocity of EDAS combined with EPS is higher than that of EDAS alone, with a lower PI in the ipsilateral artery [30]. This suggests that EDAS with EPS enhances vascular regeneration and collaboratively facilitates the regulation of intracranial artery flow [30]. These findings indicated that different revascularization procedures have different effects on cerebral hemodynamics [30]. Future investigations should explore how age‐related differences influence the molecular mechanisms underlying cerebrovascular neovascularization. Thus, subsequent studies should examine the variations in postoperative ultrasonographic parameters across different revascularization procedures to refine therapeutic strategies and improve patient outcomes.

This study also revealed that the preoperative MV in the STA had a positive correlation with the rich collateral development, respectively, when adjusted for age and Suzuki stage. This finding suggested that patients with higher preoperative MV values in the STA are likely to experience a more robust formation of collateral vessels following the procedure. This may be related to the ability of the STA to regenerate collateral circulation, such as hemodynamic demand, vascular plasticity, inflammatory mediation, etc. [33]. It suggests that the preoperative assessment of blood flow in the STA could be a predictive factor for the success of collateral circulation development. This insight could inform patient selection for surgery and guide the strategic planning of surgical procedures, potentially improving outcomes in patients with Moyamoya disease or similar cerebrovascular conditions. Further research is needed to elucidate the precise mechanisms underlying this correlation and to validate the predictive value of preoperative MV of the STA in a larger cohort.

The average age of our study cohort was 42.68 ± 10.48 years, aligning well with the recognized adult peak incidence for the MMD [34]. This targeted approach has enabled us to contribute meaningful insights into the disease's manifestations, risk factors, and potential management strategies specific to this high‐risk group. However, it is also important to acknowledge potential limitations. The age range of our study population, while representative of the peak incidence years, may not fully capture the disease's characteristics across the entire adult lifespan.

This study has several limitations. First, this study focused on hemispheres treated with a single indirect revascularization procedure with EDAS; however, currently, surgical methods for adult MMD tend to lean towards direct revascularization or direct/indirect combined revascularization surgery. Second, while this study assessed clinical events and cerebral hemodynamics using TCD, it did not include comprehensive follow‐up imaging examinations to assess cerebral perfusion and metabolism. Further exploration into the effects of revascularization surgery using Dynamic Susceptibility Contrast Magnetic Resonance Imaging (DSC‐MRI), Positron Emission Tomography (PET), or Single Photon Emission Computed Tomography (SPECT) is warranted. Third, given that MMD is a rare condition, the small sample size in our study highlights the necessity of further validation in a larger cohort. Additionally, the retrospective design may lead to selection bias, making prospective randomized controlled trials essential to confirm our findings.

## 5 Conclusions

In Moyamoya patients undergoing indirect revascularization surgery, TCD parameter findings—particularly EDV in the ipsilateral STA—correlated well with the extent of neovascularization observed on angiography. Patients developing robust collateral circulation demonstrated significant improvements in cerebral hemodynamics, as indicated by TCD, and experienced notably lower rates of cerebrovascular events. These findings highlight the critical role of ultrasound assessments, not only in monitoring postoperative outcomes but also in predicting clinical prognosis. TCD emerges as a valuable, less invasive tool for assessing collateral circulation development, providing important insights that may guide future patient management.

## Author Contributions

S.H., S.P., Y.H., X.J., C.R., S.L., and C.H.: concept and design; S.H., S.P., Y.H., J.X., L.W., H.F., X.J., and C.R.: data collection; S.H., S.P., J.X., L.W., H.F., and X.J.: data analysis; S.H., S.P., Y.H., J.X., L.W., S.L., and C.H.: interpretation of the results; S.H., S.P., Y.H., L.W., H.F., X.J., C.R., S.L., and C.H.: manuscript drafting. All authors critically revised the manuscript for important intellectual content. All authors approved the final version to be published and agreed to be held accountable for all aspects of the work.

## Consent

The requirement for written informed consent for this retrospective study was waived by the internal review board of the Fifth Medical Center of Chinese PLA General Hospital.

## Conflicts of Interest

The authors declare no conflicts of interest.

## Institutional Review Board Statement

The study received approval from the Ethics Committee of the Fifth Medical Center of Chinese PLA General Hospital (ky‐2020‐9‐22) and was conducted according to the ethical guidelines of the Declaration of Helsinki.

## Supporting information


Appendix S1.

## Data Availability

The data of this study are available on reasonable request to the corresponding author.
