# Supplementary material for: The Role of TCD in Assessing Postoperative Collateral Development and Long‐Term Clinical Outcome in Moyamoya Disease
Source: CNS Neurosci Ther. 2025 Mar 18;31(3):e70245. doi: 10.1111/cns.70245 (PMC11919775; doi:10.1111/cns.70245)
Supplement: Supplementary file 1 — Appendix S1. [file CNS-31-e70245-s001.docx]

**Supplemental material**

**Supplemental Figure 1：**Flow chart.


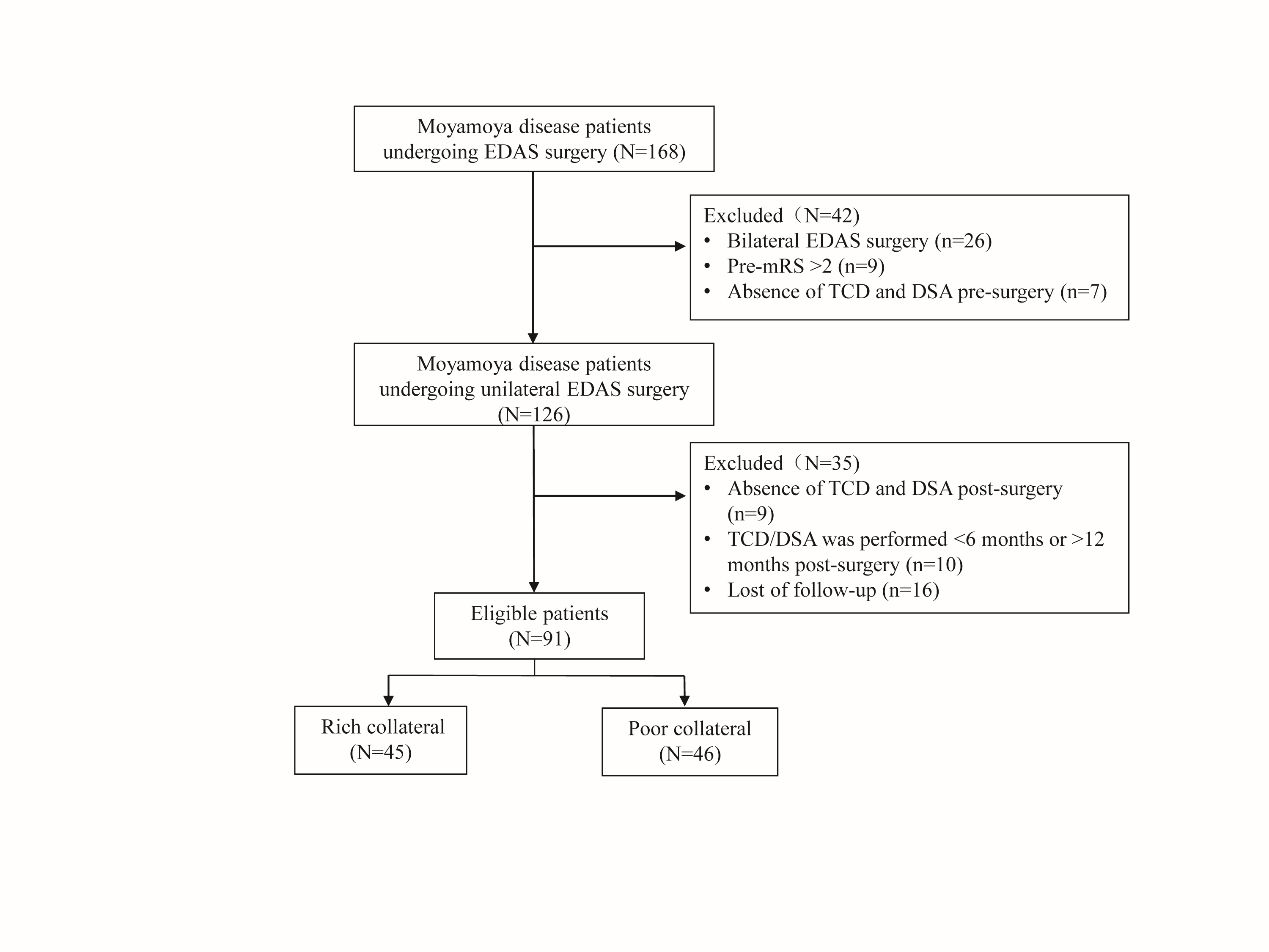


EDAS, encephaloduroarteriosynangiosis; TCD, transcranial Doppler; DSA, Digital Subtraction Angiography; mRs, Modified Rankin Scale.

**Supplementary table 1**. **The Relationship Between Preoperative STA Parameters and Collateral Development.**

|  | Univariate analysis | | Multivariate analysis* | |
| --- | --- | --- | --- | --- |
|  | *P* | OR (95%CI) | *P* | OR (95%CI) |
| Pre-STA-PSV | 0.036 | 1.04 (1.01 ~ 1.08) |  |  |
| Pre-STA-MV | 0.032 | 1.07 (1.01 ~ 1.15) | 0.032 | 1.07 (1.01 ~ 1.15) |

STA Superficial Temporal Artery, PSV peak systolic velocity, MV mean velocity, OR: Odds Ratio, CI: Confidence Interval, * Adjusted by Suzuki stages and Age.
